# Minimally Invasive Percutaneous Techniques for the Treatment of Cervical Disc Herniation: A Systematic Review and Meta-Analysis

**DOI:** 10.3390/jcm14103280

**Published:** 2025-05-08

**Authors:** Magdalena Rybaczek, Zenon Mariak, Paweł Grabala, Tomasz Łysoń

**Affiliations:** Department of Neurosurgery, Medical University of Bialystok, M. Sklodowskiej-Curie 24A, 15-276 Bialystok, Poland; zenon.mariak@umb.edu.pl (Z.M.); pgrabala@wp.pl (P.G.); lyson_t@vp.pl (T.Ł.)

**Keywords:** percutaneous, cervical, discectomy, nucleotomy, nucleoplasty, annuloplasty

## Abstract

**Background**: In recent decades, the adoption of minimally invasive (non-endoscopic) cervical techniques has grown significantly. Advancements in surgical instrumentation have broadened the spectrum of available percutaneous interventions, thus providing viable alternative treatment options for patients with prolonged, conservative treatment-resistant ailments due to contained cervical disc herniation. The aim of this study was to perform a systematic review and meta-analysis in order to evaluate the effectiveness and safety of minimally invasive percutaneous (non-endoscopic) cervical techniques. **Methods**: A comprehensive literature search was conducted using the PubMed, Cochrane Library, and SCOPUS databases up to July 2024, in accordance with the PRISMA guidelines. Outcomes measured included Visual Analogue Scale (VAS) scores, the Neck Disability Index (NDI), and MacNab scores, assessing pain relief and functional recovery. The risk of bias was evaluated using the Cochrane risk of bias tool (RoB 2) and the risk of bias in nonrandomized studies of interventions (ROBINS-I) tool, with statistical analyses conducted in R software (version 4.3.1). **Results**: Out of 847 records, 21 studies (covering 1580 patients) were included in the final analysis. Five different percutaneous minimally invasive cervical procedures were incorporated into this review: nucleoplasty (*n* = 973), discectomy (*n* = 311), a combination of nucleoplasty and discectomy (*n* = 98), annuloplasty (*n* = 33), and pulsed radiofrequency (*n* = 17). The mean patient age was 49.5, with a gender distribution of 47.7% male and 52.3% female. A meta-analysis of six studies on cervical nucleoplasty (400 patients) demonstrated a significant reduction in pain scores, with a standardized mean difference (SMD) of −4.68 (95% CI: −8.77; −0.59, *p* = 0.032). However, a high heterogeneity (I^2^ = 98.8%, Q = 407.31, *p* < 0.001) was observed, indicating significant variability across studies. The reoperation rate among patients was 3.4%, with discitis and device-related complications being the most frequently reported adverse events. **Conclusions**: Minimally invasive percutaneous cervical interventions provide effective pain relief and functional improvement for patients with cervical disc herniation, as evidenced by reductions in VAS scores and positive MacNab outcomes. The choice of the most appropriate technique should be based on individual clinical scenarios, surgeon expertise, and patient preferences, as no single method demonstrates clear superiority according to clinical outcomes or complication rates.

## 1. Introduction

Cervical radiculopathy is a prevalent neurological condition affecting the world population. It is typically characterized by neck pain radiating to the arm, muscle weakness, and sensory paresthesia, resulting from nerve root dysfunction, often due to mechanical compression. When prolonged conservative management proves ineffective and magnetic resonance imaging (MRI) findings do not indicate the need for traditional open surgery, such as anterior cervical discectomy and fusion (ACDF), minimally invasive (non-endoscopic) percutaneous techniques present a viable alternative treatment option. In recent decades, the adoption of percutaneous techniques has grown significantly, with advancements in instrumentation broadening the spectrum of available interventions. Among these approaches, three procedures have attracted particular attention: percutaneous discectomy, nucleoplasty, and annuloplasty. These techniques can be applied separately or combined, offering different modes of action in the cervical intervertebral disc.

Although numerous studies have reported their clinical success, inconsistencies in study design, such as varying patient selection criteria and limited follow-up periods, make it difficult to assess their actual effectiveness. We aim to conduct a literature review and meta-analysis of cervical minimally invasive percutaneous (non-endoscopic) procedures in order to evaluate their clinical efficacy and safety.

While this systematic review encompasses a broad range of minimally invasive percutaneous techniques for cervical disc herniation, the meta-analysis component was limited exclusively to percutaneous cervical nucleoplasty (PCN) due to insufficient and heterogeneous data available for other techniques.

## 2. Materials and Methods

The presented systematic review and meta-analysis were performed according to the Preferred Reporting Items for Systematic Reviews and Meta-Analysis (PRISMA) guidelines (Supplementary Materials).

### 2.1. Search Strategy

**Figure 1:**
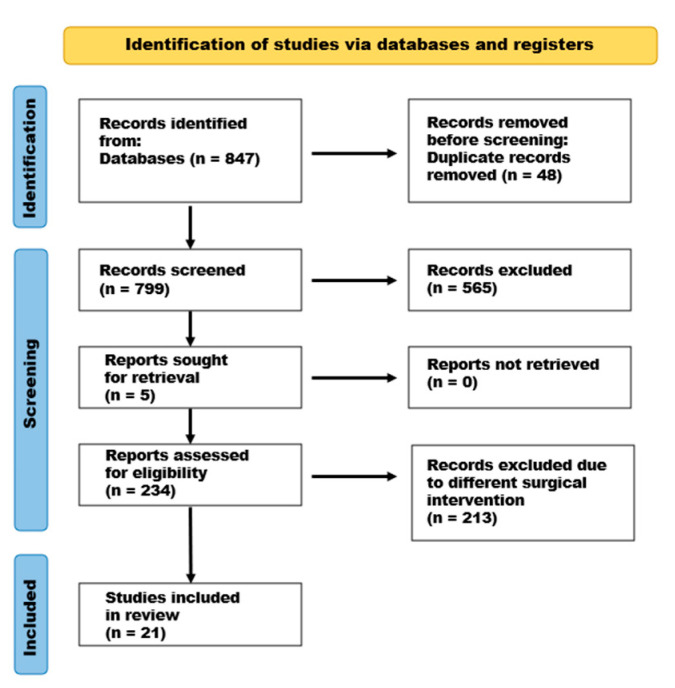
PRISMA flow diagram depicting the selection of the studies included in this review.

The PubMed, Cochrane Library, and SCOPUS databases were searched using the term “percutaneous”, in combination with “cervical nucleotomy”, “cervical discectomy”, “cervical nucleoplasty”, and “cervical annuloplasty”, up to July 2024. A schematic PRISMA flow diagram depicting the selection of the studies included in this review is presented in Figure 1.

### 2.2. Study Selection and Data Extraction

Two authors independently screened the literature by reading the full text of manuscripts to extract information on the authors and publication year; country; characteristics of participants; type of intervention and qualification scheme; and effectiveness outcomes measured with clinical scales, such as the Visual Analogue Scale (VAS), McNab score, Neck Disability Index scale (NDI).

### 2.3. Inclusion Criteria

The inclusion criteria, based on the PICOS framework, were as follows:

P (patients): Patients with cervicogenic ailments and diagnosed with contained cervical disc herniation (protrusions without rupture of the annulus fibrosus with no evidence of extrusion or sequestration) during MRI examination.
I (intervention): Patients who underwent percutaneous, minimally invasive cervical spine interventions.
C (comparison): Individuals who underwent other cervical spinal interventions and those with cervicogenic ailments who were treated conservatively.
O (outcomes): Results obtained with minimally invasive interventions or via patient’s observations, with the VAS assessment, NDI score, and MacNab scale used as indicators of treatment success during various follow-up periods. Both complications and re-surgery rates were also reviewed.
S (study design): All research articles, case studies, and comparative studies such as case–control studies, cohort studies, and randomized clinical trials published in English up to July 2024 were taken into consideration. The included studies were required to report at least one of the above outcomes.

### 2.4. Exclusion Criteria

Patients who underwent endoscopic cervical spine interventions or anterior cervical spine decompression and fusion (ACDF) were excluded from the review. We also excluded articles concerning laser treatment, the implementation of implants/artificial discs (GelStix, Discogel, HydroGel), or cervical spine stabilization.

### 2.5. Data Items

We extracted the following data from each study: the first author’s name, year of publication, country, study design, number of participants, age and sex of participants, type of percutaneous procedure, spinal levels of interventions, scales used for outcome assessment, complications and reoperation rates, and follow-up duration.

### 2.6. Data Availability

Although our initial objective was to perform a meta-analysis across all identified percutaneous techniques, significant variability in outcome reporting and lack of essential statistical data restricted the quantitative synthesis to studies evaluating percutaneous cervical nucleoplasty (PCN).

### 2.7. Risk of Bias Assessment

Two authors (M.R. and T.L.) independently assessed the risk of bias in the included studies using the 18 March 2021 version of the Cochrane risk of bias tool for randomized trials (RoB 2) and the risk of bias in nonrandomized studies of interventions (ROBINS-I) tool for RCT and nonrandomized studies. In case of any assessment discrepancies, the senior author (Z.M.) adjudicated.

### 2.8. Statistical Analysis

The studies included in this research and the available information regarding patients’ satisfaction with percutaneous treatment allowed for the conduction of a meta-analysis focused solely on percutaneous cervical nucleoplasty. The primary outcome was the dynamics of pain relief, measured using the VAS. The treatment effect was assessed by comparing the differences in pain levels mainly recorded at the long-term follow-up (FU) with those recorded at baseline (prior to the procedure). Pain severity was quantitatively assessed at two distinct temporal junctures using a standardized scale, employing a longitudinal design within a single cohort. The initial measure of pain severity was recorded pre-operatively, serving as a baseline comparator, while subsequent assessment occurred post-operatively, primarily during extended follow-up periods. This methodological approach enabled the isolation of the intervention’s effect on pain reduction over time, facilitating a rigorous analysis of percutaneous nucleoplasty as a therapeutic intervention for managing disc-related pain.

To estimate the pooled effect size, a random effects model with a restricted maximum likelihood (REML) estimator was employed, utilizing the inverse variance method [1]. This approach was selected to accommodate the expected heterogeneity among the individual study results, allowing for a more nuanced aggregation of data that acknowledged variations in study design and population characteristics. The Hartung–Knapp adjustment based on a t distribution, as delineated by Hartung and Knapp, was applied to effectively address the random effects in this analysis, providing a more conservative estimate of the true effect size in the context of significant observed heterogeneity [2]. Additionally, Hedges’ g, a bias-corrected standardized mean difference, was also utilized to ensure that the estimation of the effect size was accurately adjusted for small-sample biases. The assessment of between-study heterogeneity was meticulously conducted using several statistical measures: I^2^, τ^2^, τ, and H statistics [3,4]. These indices provided a comprehensive evaluation of the variations across studies. Additionally, Cochran’s Q test was employed to further quantify the degree of heterogeneity [5]. The threshold for statistical significance was established at an alpha level of α = 0.05. Numerical variables are summarized using the mean (M) and standard deviation (SD) and, in certain instances, range. Categorical variables are summarized using counts (*n*) and percentages (%). Analyses were conducted using the R Statistical language (version 4.3.1; R Core Team, 2023) on Windows 10 pro 64 bit (build 19045), using the packages meta (version 6.5.0), dmetar (version 0.1.0), esc (version 0.5.1), report (version 0.5.7), and dplyr (version 1.1.3).

## 3. Results

### 3.1. Study Selection

A search of the manuscripts related to our review, in accordance with the PRISMA methodology, resulted in 847 records: 19 related to “percutaneous cervical nucleotomy”, 708 related to “percutaneous cervical discectomy”, 114 related to “percutaneous cervical nucleoplasty”, and 6 related to “percutaneous cervical annuloplasty”. After 48 duplicate records were removed, the studies were screened by title and abstract, and 565 were excluded due to unrelated subject matter. Then, we reviewed the full text of the remaining 234 studies, of which 213 were excluded due to the use of endoscopic interventions, laser treatment, intervertebral implants/artificial discs, or cervical stabilization surgery. The full-text detailed inspection resulted in the inclusion of 21 articles [6,7,8,9,10,11,12,13,14,15,16,17,18,19,20,21,22,23,24,25,26]. A PRISMA flow diagram of the study selection process is presented in Figure 1.

The 21 included studies were published between 2005 and 2022 and were conducted in China (*n* = 6), Italy (*n* = 5), the Netherlands (*n* = 6), Germany (*n* = 3), Korea (*n* = 2), and Taiwan (*n* = 1). Five different minimally invasive cervical procedures were incorporated into this review, and the number of performed surgical interventions was as follows: percutaneous cervical nucleoplasty, 973; percutaneous cervical discectomy, 311; percutaneous cervical nucleoplasty and discectomy, 98; percutaneous cervical annuloplasty, 33; and percutaneous pulsed radiofrequency, 17.

The included studies comprised 1580 patients, of whom 1432 underwent cervical percutaneous intervention, and 124 were treated conservatively. Due to the inclusion of comparative studies, 24 patients from control groups underwent anterior cervical discectomy (ACD). Among the operated patients, 696 were male (47.7%), and 713 were female (52.3%), of whom the weighted average age was 49.5 (based on data available in 19 studies). Among the patients treated conservatively, 46 were male (37.1%), and 58 were female (62.9%). The total number of intervertebral disc levels treated in interventions was 1624: C2/C3 (*n* = 2), C3/C4 (*n* = 121), C4/C5 (*n* = 324), C5/C6 (*n* = 698), C6/C7 (*n* = 477), and C7/Th1 (*n* = 2).

The mean follow-up ranged from 2 months to 6 years. Table 1 presents the study characteristics and outcomes of the systematic review.

### 3.2. Risk of Bias Assessment

Following the protocols of the Cochrane risk of bias tool for randomized trials (RoB 2), we assessed five RCTs. The 16 remaining studies had prospective (*n* = 6) and retrospective (*n* = 10) case series designs, and they were assessed using the risk of bias in nonrandomized studies of interventions (ROBINS-I) tool (Table 2 and Table 3).

### 3.3. Percutaneous Cervical Nucleoplasty—Meta-Analysis and Outcomes

A meta-analysis encompassing six studies (k = 6), with a total of 400 observations, provided significant insights into the efficacy of percutaneous nucleoplasty in managing disc-related pathologies. The pooled results show a substantial standardized mean difference (SMD) of −4.68. The confidence interval (CI 95%: −8.77; −0.59), along with t = −2.94 and *p* = 0.032, supports the conclusion that percutaneous nucleoplasty significantly reduces symptoms compared to control (pre-operation) conditions, although the effect varies widely across studies.

**Figure 2:**
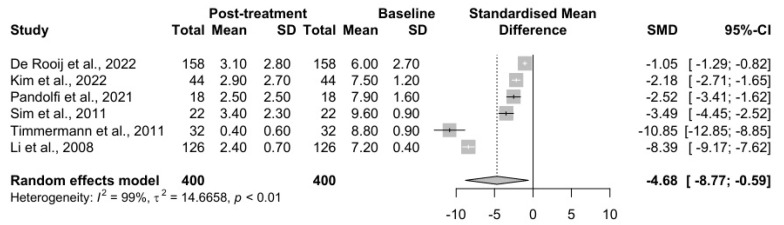
Comprehensive visualization of individual studies and the overall treatment efficacy of percutaneous nucleoplasty in patients with cervical discopathy. SD—standard deviation; SMD—standardized mean difference [6,7,8,11,12,13].

The heterogeneity quantified in this analysis is exceptionally high, with I^2^ = 98.8%, indicating that 98.8% of the variability in effect sizes is due to heterogeneity rather than chance. This is further corroborated by a very high H = 9.03, suggesting substantial variability among the included studies. The tau-squared value, τ^2^ = 14.67, with its associated τ = 3.83, reflects this high dispersion, supported by their respective confidence intervals. The Q-test for heterogeneity is highly significant (Q = 407.31, df = 5, *p* < 0.001), confirming that the effect sizes vary significantly across the studies included in the meta-analysis. A graphical depiction of the effect results, which facilitates a comprehensive comparison across various studies, is presented using a forest plot in Figure 2.

To address the high heterogeneity observed in the meta-analysis (I^2^ = 98.8%), a subgroup analysis was conducted based on study design and follow-up duration. The studies were categorized into two groups: short-term follow-up (less than 12 months) and long-term follow-up (12 months or more). The results are presented in Table 4.

**Figure 3:**
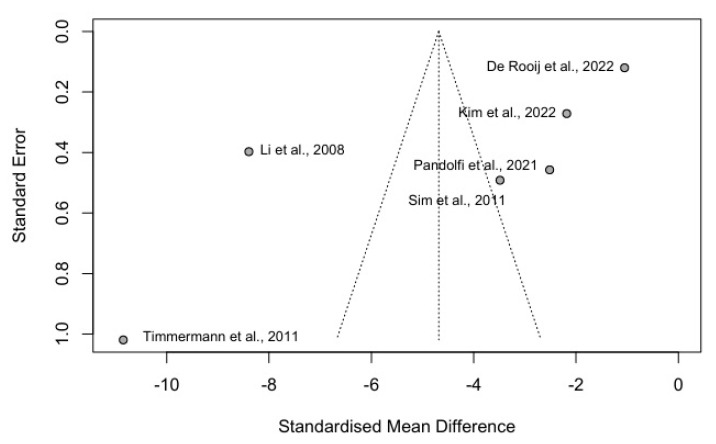
Funnel plot illustrating the distribution of individual studies, represented by standardized mean difference (SMD) and standard error (SE) values [6,7,8,11,12,13].

The findings of the publication bias analysis are depicted in a funnel plot, illustrated in Figure 3.

The results of the linear regression test of the funnel plot asymmetry, obtained using the Pustejovsky method, provide insights into the distribution of study effects in the meta-analysis. The test result, based on a weighted linear regression of the treatment effect on the square root of the sum of the inverse group sample sizes using the treatment effect variance as weights, showing t = −0.62 and *p* = 0.570, strongly suggests that there is no significant asymmetry in the funnel plot. This outcome indicates a lower likelihood of publication bias or other types of small-study effects under this analysis method compared to Egger’s test (which was also nonsignificant but only at a trend level, *p* = 0.064).

### 3.4. Literature Review

#### 3.4.1. Minimally Invasive Percutaneous Surgical Techniques Used Among the Studies

##### Percutaneous Cervical Nucleoplasty (PCN)

Percutaneous cervical nucleoplasty is a minimally invasive, image-guided procedure intended to decompress the intervertebral disc by removing or ablating nucleus pulposus tissue using radiofrequency technology. During the procedure, the patient is positioned supine with the neck slightly extended. A rolled towel or neck roll may be placed under the shoulders to enhance exposure of the cervical spine. The procedure is generally performed under local anesthesia. The trajectory for the procedure is planned through an anterolateral approach, ensuring that the carotid artery, jugular vein, trachea, and esophagus are avoided. An introducer cannula, typically sized between 19G and 21G, is advanced to the targeted disc level, with confirmation using both anteroposterior and lateral X-ray views. Once the needle tip is verified to be in the nucleus under lateral and AP fluoroscopy, a radiofrequency coblation probe (such as the SpineWand) is introduced through the needle. The probe is then activated to create four to six radial ablation channels in a starburst or spoke-wheel pattern, with each channel lasting 10 to 15 s. This process generates micro-channels and decompression zones within the nucleus, applying a temperature range of approximately 40 to 70 °C. After the ablation is complete, the instruments are withdrawn, and a sterile dressing is applied [6,7,8,9,10,13,21,24].

##### Percutaneous Cervical Discectomy (PCD)

Percutaneous cervical discectomy is a minimally invasive procedure aimed at decreasing pressure in the intervertebral disc space by removing nucleus pulposus material from the cervical disc. During the procedure, the patient is positioned supine with a slight extension of the neck to facilitate access to the anterior cervical spine. Standard sterile preparation and draping are performed prior to the procedure. Conscious sedation or local anesthesia is typically used. Under X-ray fluoroscopic guidance, specifically in both anteroposterior and lateral views, a percutaneous anterolateral approach is utilized. A small skin incision of approximately 5 mm is made, through which a guide needle is introduced via the anterior neck to reach the intervertebral disc, carefully navigating between vascular and visceral structures to ensure safety. Using anatomical landmarks, a spinal needle (generally 18-gauge) is advanced toward the affected disc space, all while avoiding the carotid sheath and the trachea/esophagus. Correct placement of the needle into the nucleus pulposus is confirmed through X-ray scans. Next, a dilator and a working cannula are inserted over the guide needle to establish access. Through the cannula, forceps or rongeurs are employed to extract disc fragments. A small volume of nucleus pulposus material is removed, which helps to reduce intradiscal pressure and alleviate nerve root compression. The amount of disc material removed is carefully limited to prevent disc collapse or segmental instability. Once the procedure is complete, the tools are removed and the incision is closed with a sterile strip [12,14,16,18,19,20].

##### Percutaneous Cervical Annuloplasty (PCA)

Percutaneous cervical annuloplasty is a minimally invasive procedure designed to treat cervical discogenic pain that arises from annular tears or internal disc disruption. This technique involves the targeted application of thermal modulation or coagulation to the posterior annulus fibrosus using radiofrequency energy, which helps to denervate pain-sensitive nerve endings. The procedure is performed with the patient in a supine position under local anesthesia. To facilitate neck extension, a bolster is placed beneath the shoulders. Continuous verbal communication is maintained to monitor neurological function throughout the intervention. Fluoroscopy is employed to accurately target the posterior or posterolateral annulus. A 19-gauge introducer needle or cannula is carefully advanced to the posterior third of the disc, just anterior to the posterior annular margin, while avoiding critical neurovascular structures such as the carotid sheath and the tracheoesophageal complex. A radiofrequency probe, such as a coblation wand, is then inserted into the annular tear or the area of high signal intensity identified on prior MRI scans (commonly referred to as the high-intensity zone, HIZ). Controlled radiofrequency energy, set at temperatures between 40 and 60 °C, is applied circumferentially to the annular region. This process causes denervation and collagen shrinkage. Typically, several cycles of ablation are performed, each lasting about 10 s, with the probe rotated to ensure coverage of the symptomatic annular zone. After the ablation, the instruments are removed and the incision is closed with a sterile strip [8,14,15].

##### Percutaneous Pulsed Radiofrequency (PRF)

Percutaneous pulsed radiofrequency of the cervical dorsal root ganglion is a neuromodulatory procedure that delivers intermittent, non-destructive radiofrequency (RF) energy to the dorsal root ganglia involved in radicular pain. The patient is positioned supine or in a slight lateral decubitus position, depending on the preferred approach. Under local anesthesia, a radiofrequency cannula (typically 22G) is inserted into the intervertebral foramen, targeting the dorsal root ganglion (DRG) at the affected level. The procedure is monitored using X-ray fluoroscopy. Motor and sensory stimulation at frequencies of 50 Hz and 2 Hz is used to confirm correct needle placement near the DRG without causing motor symptoms. Radiofrequency pulsing is applied at a frequency of 2 Hz with a pulse width of 20 milliseconds for 120 s per cycle, typically performed for two cycles.

The temperature is maintained below 42 °C to prevent tissue destruction [25].

##### Anterior Cervical Discectomy (ACD)

The primary goal of this technique is to decompress the affected cervical nerve roots by removing the intervertebral disc and/or disc herniation that is compressing the nerve root.

The patient is placed in the supine position under general anesthesia. The head is positioned in slight extension to optimize access to the anterior cervical spine. A shoulder roll may be used to enhance neck extension and maintain a straight cervical spine. A small horizontal incision (approximately 2–3 cm) is made over the anterior aspect of the cervical spine, typically at the level of the affected disc. The incision is made along natural skin folds to minimize scarring. The skin and subcutaneous tissues are carefully dissected to expose the platysma muscle. The sternocleidomastoid muscle is retracted laterally, and the carotid sheath containing the carotid artery, internal jugular vein, and vagus nerve is gently displaced laterally to provide better access. The trachea and esophagus are carefully avoided during the approach. The prevertebral fascia is opened, and the anterior longitudinal ligament (ALL) is identified and incised to expose the cervical disc. Cervical disc material is removed using specialized instruments such as a disc rongeur, pituitary rongeur, or curette. The herniated disc material is excised, and the posterior portion of the disc is often removed to relieve nerve root compression. The fascia, muscle layers, and skin are carefully closed in layers. A sterile dressing is applied [26].

##### Indications and Contraindications for Minimally Invasive Percutaneous Surgical Techniques

All indications and contraindications are presented in Table 5.

### 3.5. Results of the Literature Review

#### 3.5.1. Percutaneous Cervical Nucleoplasty (PCN) vs. Percutaneous Cervical Discectomy (PCD)

A study conducted by Ierardi et al. in 50 patients (26 PCN and 24 PCD), with follow-ups at 2 and 6 months, found no significant differences in clinical outcomes or complications between the two techniques [16]. Results assessed using the McNab scale indicated a similar efficacy of the two methods: After 2 months, 22 out of 26 PCN patients and 20 out of 24 PCD patients scored “excellent/good”. After 6 months, 21 out of 26 PCN and 19 out of 24 PCD patients had the same results. No complications were reported.

Yan et al. conducted a study on a larger group of 176 patients (81 PCN and 95 PCD), with a longer mean follow-up period of 29 months (16–48 months). They also found no significant differences in clinical outcomes or spinal stability between the two techniques [20]. Results assessed using the McNab scale showed that the percentage of “excellent/good” outcomes was 77.8% for PCN and 79.5% for PCD. Both PCN and PCD patients experienced a significant improvement in their pain index, which decreased from 7.1 ± 1.1 to 2.7 ± 0.9 in both groups. A Perc-D Spine Wand broke in the C4/C5 disc space after PCN, and one case of discitis was reported as a complication of PCD.

#### 3.5.2. PCN + PCD, PCD vs. PCN vs. PDCN, PCD

In a study by Li et al., the efficacy of combined PDCN (PCN + PCD) was evaluated in 74 patients with vertigo caused by cervical disc herniation [17]. The average effectiveness of the treatment was 94.6% after one week and 90.6% after at least one year of follow-up. Only two cases of discitis were reported as complications, resulting in a complication rate of 2.7%. The results suggest that PDCN is an effective method that improves both early and late clinical outcomes, especially in terms of improving the function of patients with vertigo. Another study by Yang et al. compared three techniques, namely, PCD, PCN, and PDCN, in a sample of 171 patients [18]. The MacNab scores were similar in all groups, with excellent or good results obtained in 81.3% of patients after PCD, in 82.4% after PCN, and in 83.2% after PDCN. The study reported complications, such as a plasma knife breaking during PCN and discitis after the PCD and PCN procedures. However, neither method showed an advantage in terms of the risk of spinal instability after surgery.

Schubert and colleagues focused on patients undergoing PCD, analyzing their health status two years after the procedure [19]. The results showed that 81.4% of patients had excellent or good results according to the MacNab scale and that 94.2% would choose the same procedure again. Despite nine reoperations over the two years, the authors found PCD to be a safe and effective treatment for cervical radiculopathy. Across the reviewed studies, the total effectiveness of these techniques, based on the McNab excellent/good ratings, ranged from 75.7% to 85.1%. The average McNab excellent/good rating was approximately 81.6% for PCN, 81.9% for PCD, and 79.5% for PCDN. Both PCN and PCD demonstrated significant improvements in VAS scores, with reductions from approximately 7 to 2.7. The complications reported in these studies indicate that discitis has the highest complication rate at 2.7%, observed after the PCDN procedure.

#### 3.5.3. Percutaneous Cervical Nucleoplasty (PCN) vs. Anterior Cervical Discectomy (ACD)

In a randomized, controlled study conducted by Rooij et al. [26], 48 patients were enrolled, with 24 patients undergoing PCN and the remaining 24 receiving ACD [26]. After a follow-up period of 12 months, a slight improvement in shoulder pain was observed in the ACD group. However, this improvement was not statistically significant. No serious adverse events were reported in either group.

#### 3.5.4. Percutaneous Cervical Nucleoplasty (PCN) vs. Conservative Treatment (CT)

Chen et al. conducted a study involving 92 patients, with 71 receiving PCN and 21 undergoing CT [21]. The results demonstrated that PCN was significantly more effective than CT in terms of pain relief and functional improvement, as measured using VAS, ODI, and NDI scores during the first three months post-treatment. Although a reduction in disc height was observed following PCN, the authors noticed that it did not directly correlate with clinical outcomes, suggesting a more complex mechanism of improvement. Notably, the reoperation rate was approximately 4.44% (4/90) within an average of 30.44 ± 24.36 months after surgery, indicating stable medium-term outcomes for PCN. Cesaroni et al. evaluated the outcomes of PCN versus CT in 115 patients over a one-year follow-up period [22]. Significant improvements in VAS scores were observed in the PCN group compared to in the CT group. Additionally, scores on the NDI and Short Form-36 (SF-36) scales favored PCN, further highlighting its advantage. The absence of complications and the sustained functional improvement over the year suggest that PCN is both an effective and safe treatment, offering long-term stable outcomes. In a two-year follow-up study by Birnbaum involving 56 patients, PCN demonstrated superior efficacy to CT in terms of pain reduction at both 12 and 24 months [23]. The results emphasized the durability of PCN’s effects, with a notable lack of complications. The significant reduction in pain and the improved outcomes in the PCN group further confirmed its superiority over conservative treatment. Nardi et al. compared the short-term results of PCN and CT in 70 patients over a 60-day follow-up period [24]. In the PCN group, 80% of patients experienced complete symptom resolution, markedly outperforming the CT group, where 75% of patients reported persistent pain. Additionally, patients treated with PCN returned to work more quickly, demonstrating faster functional recovery. These results were statistically significant, underscoring the efficacy of PCN in the short term.

#### 3.5.5. Percutaneous Cervical Nucleoplasty (PCN) vs. Percutaneous Pulsed Radiofrequency (PRF)

Halim et al. compared PCN with PRF in the treatment of cervical intervertebral disc herniation [25]. The study involved 34 patients in a prospective, randomized clinical trial. After three months of follow-up, the average improvement in the VAS score (measured from 0 to 100) was 43.4 points in the PCN group and 34.0 points in the PRF group. Patient satisfaction with the treatment results was higher in the PRF group (63.5 points) than in the PCN group (58.4 points), but the difference was not statistically significant (*p* = 0.69). At three months, both PCN and PRF resulted in a significant reduction in pain, without one method showing superiority over the other.

#### 3.5.6. Percutaneous Cervical Annuloplasty (PCA)

He et al. presented the results of PCA among 33 patients with cervicogenic neck pain without shoulder radiculopathy [15]. This was a prospective observational study. After one year of follow-up, there was a significant improvement in the VAS score, where the mean score decreased from 6.8 ± 0.9 before surgery to 2.5 ± 1.3 after surgery.

#### 3.5.7. Complications and Reoperation Rate

A systematic review revealed that intervertebral discitis was the most common complication of percutaneous procedures and occurred in six cases—in two cases after PCN [14,18], in two cases after PCD [18,20], and in two cases after PDCN [17]. These findings indicate that discitis, although relatively rare, is associated predominantly with percutaneous techniques, particularly **PCN**, **PCD**. This underscores the necessity of strict adherence to aseptic techniques during these procedures, as well as vigilant postoperative monitoring to ensure early detection and management of infections.

Adverse events related to surgical devices consisted of a broken Perc-D Spine Wand in three cases and a broken plasma knife in one case during percutaneous cervical nucleoplasty [13,14,18,20]. These complications were exclusively linked to the **PCN** technique. These incidents highlight the importance of using high-quality, reliable surgical instruments and suggest that surgeon experience plays a key role in minimizing technical complications.

The need for **reoperation** represents an important aspect of the safety and long-term efficacy profile of percutaneous cervical procedures. The general reoperation rate reported in six studies was 10.4% (49/471 patients).

In terms of separate estimations, the rate after percutaneous cervical nucleoplasty was assessed as 10.6% (40/376 patients) [6,7,9,21,24], and the rate after percutaneous cervical discectomy was assessed as 9.5% (9/95 patients) [19]. Based on the conducted review, the total reoperation rate among patients who underwent a minimally invasive percutaneous cervical intervention was 3.4% (49/1432 patients).

## 4. Discussion

### 4.1. Meta-Analysis Outcomes

Percutaneous cervical nucleoplasty was the most common intervention presented in this review, with a satisfactory effect estimated as 52.9–93.3%. However, the meta-analysis results raise some considerations. Sample sizes across the studies vary considerably, ranging from as few as 18 participants to as many as 158. This discrepancy in sample size impacts not only the reliability of the results but also their applicability to broader populations, with larger samples typically providing more robust data that may be considered more generalizable. Gender distribution also varies widely among the studies, from a low of 21.9% to a high of 68.2% of male participants. This variation highlights potential differences in the prevalence of cervical discopathy among genders or possibly different responses to treatment. The duration of follow-up in these studies also shows significant variability, ranging from a short period of 1 month to as long as 41.5 months. Longer follow-ups, such as in the study by De Rooij et al. [6], are invaluable for assessing the long-term effectiveness and safety of treatments, whereas shorter follow-ups (Timmermann et al. [12]) may prioritize immediate outcomes without capturing the long-term effects, as shown in Table 6.

While the results suggest a positive effect of percutaneous nucleoplasty on symptom reduction, the high levels of heterogeneity indicate that the effect is not consistent across all study settings or patient populations. To address the high heterogeneity observed in the original meta-analysis (I^2^ = 98.8%), we decided to perform a subgroup analysis shown in Table 4. Studies with short-term follow-up (<12 months) consistently demonstrated very large effect sizes, indicating substantial pain relief shortly after the procedure. This suggests that PCN is highly effective in providing rapid symptom control for patients with contained cervical disc herniation. Both Sim et al. (2011) [11] and Timmermann et al. (2011) [12] reported effect sizes exceeding 2.9, which corresponds to a strong and clinically meaningful reduction in pain.

In contrast, studies with long-term follow-up (≥12 months) showed greater variability in treatment effects. While some studies, such as Kim et al. (2022) [7] and Pandolfi et al. (2021) [8], maintained moderate-to-large benefits over time, others, like De Rooij et al. (2022) [6], reported minimal sustained improvement. Notably, Li et al. (2020) [9] documented a negative effect size, suggesting a potential worsening of symptoms or recurrence.

These findings highlight that while PCN can be considered an effective short-term intervention, its long-term efficacy remains inconsistent across studies.

The subgroup analysis demonstrated a notable difference in treatment effect sizes between prospective and retrospective studies. Prospective studies showed a significantly larger pooled effect (Hedges’ g = 3.28), indicating substantial clinical benefit of percutaneous cervical nucleoplasty (PCN). In contrast, retrospective studies reported a minimal effect (Hedges’ g = 0.07), suggesting limited observed efficacy.

This variation likely reflects the methodological strengths of prospective designs, which typically ensure standardized patient selection, consistent outcome measurement, and structured follow-up protocols. These factors reduce bias and enhance the reliability of reported outcomes. Conversely, retrospective studies are more susceptible to selection bias, incomplete data, and heterogeneity in clinical practice, all of which may contribute to the underestimation of treatment effects.

According to the results in Figure 3, there appears to be some degree of asymmetry in the distribution. The studies with the largest negative effects also exhibit larger standard errors, which could suggest a possible publication bias or other systematic differences among the studies (particularly with Timmermann et al. [12] and Li et al. [13]). This pattern could indicate that larger, possibly more variable studies or studies with more extreme outcomes are more likely to be published.

The calculated bias of −9.21, although negative, comes with a large standard error of SE = 14.88, which undermines the precision and reliability of this estimate. Similarly, the negative intercept at −0.59, with an even larger standard error of SE = 2.39, does not provide strong evidence of any systematic bias across the studies. These large standard errors reflect substantial uncertainty in the measurement of both the bias and intercept, suggesting that any real effect, if present, is difficult to detect with confidence in this dataset.

Additionally, the very high multiplicative residual heterogeneity variance (τ^2^ = 92.92) points to significant variation among the study effects that cannot be explained solely by sampling variability. The predictor used in this analysis is intended to provide a more sensitive assessment of asymmetry in contexts with high heterogeneity.

### 4.2. Risk of Bias and Methodological Considerations

The risk of bias assessments conducted using ROBINS-I and RoB 2 tools revealed important insights into the methodological quality of the included studies. Most non-randomized studies were evaluated using the ROBINS-I tool, which highlighted moderate to serious risk of bias in several domains, particularly in the measurement of outcomes, selective reporting, and missing data. These concerns are relevant considering that many studies relied on subjective outcome measures (such as the VAS), often without clearly defined or consistent follow-up intervals.

Notably, studies that demonstrated higher methodological quality (i.e., prospective design with low bias) tended to report substantially larger treatment effects. Our subgroup analysis (Table 4) confirmed this pattern: prospective studies showed a much higher pooled effect size (Hedges’ g = 3.28; 95% CI: 2.44–4.11) compared to retrospective studies (Hedges’ g = 0.07; 95% CI: −0.07 to 0.21). This suggests that methodological rigor may significantly influence outcome estimates in studies of percutaneous cervical nucleoplasty.

Furthermore, randomized controlled trials assessed via the RoB 2 tool showed a predominantly low risk of bias. However, two trials had some concerns, primarily related to participant recruitment (Nardi 2005 [24]) and randomization processes (Rooij 2020 [26]). These observations further underscore the variability in study quality and its potential contribution to the high heterogeneity observed in our meta-analysis.

### 4.3. Limitations

We are aware that our study has some limitations. The analysis of available studies on percutaneous techniques for cervical disc herniation, including PCN, PCD, or a combination of both, reveals significant heterogeneity in patient populations and selection criteria. The variability of results depends on the studied populations and individual patient factors, such as age, health condition, and level of physical activity, which may affect the final effect of the therapy. Poorly defined qualification criteria concerning the level of cervical herniation progression on radiological imaging may significantly affect the results and reduce the comparability across studies. These methodological inaccuracies hinder the ability to draw definitive conclusions about the precise effectiveness of these procedures.

Despite these methodological challenges, the evidence indicates that both PCN and PCD techniques are safe and effective. Studies have reported low complication rates, with only a few adverse events, such as discitis or technical device problems. This favorable safety profile is a significant advantage of percutaneous approaches over more invasive surgical techniques.

Comparisons of PCN and conservative treatment show that PCN provides significantly better pain relief, improved function, and a faster return to daily activities. The observed benefits of PCN over conservative approaches are consistent with findings from long-term follow-up studies. Interestingly, PCN was assessed as a viable alternative to ACD for treating cervical disc herniation.

The duration of follow-up periods differed widely across studies; however, long-term evaluations consistently showed that minimally invasive percutaneous techniques were effective and led to high levels of patient satisfaction. These results highlight the potential of these techniques to offer lasting relief from symptoms related to cervical disc herniation. They could bridge the gap between prolonged, ineffective conservative treatment and the need for traditional open ACDF.

The McNab score for percutaneous cervical annuloplasty showed that 64% to 82% of patients rated the procedure as “excellent” or “good” at one-year follow-up. This suggests a relatively high effectiveness in the short term. However, the paucity of literature prevents a reliable assessment of the long-term efficacy, clinical usefulness, and complication rates of this procedure. While the current results are promising, they are based on a relatively small sample size, which impacts the overall reliability of the data.

As a future direction, it would be insightful to compare the results of minimally invasive percutaneous (non-endoscopic) cervical techniques with those of “traditional” surgeries. This comparison could help determine whether minimally invasive approaches provide significant benefits that could potentially delay or, in some cases, even avoid the need for ACDF surgery.

## 5. Conclusions

Minimally invasive percutaneous cervical techniques represent an effective treatment option for cervical disc herniation, demonstrating favorable outcomes in terms of pain relief and functional recovery. However, the interpretation of these results is limited by significant heterogeneity across studies, small sample sizes, and a lack of robust long-term comparative data. These factors reduce the ability to draw definitive conclusions regarding the superiority or durability of these interventions.

Percutaneous cervical procedures are generally safe; however, complications such as discitis, instrument breakage, and the need for reoperation, although infrequent, should be carefully considered in the risk assessment. Emphasizing proper surgical protocols, equipment standards, and patient selection criteria is essential to further enhance procedural safety. Current evidence supports the use of percutaneous techniques in selected patients, but further large-scale, randomized, and long-term studies are necessary to validate these findings and better define their role in clinical practice.

## Figures and Tables

**Table 1:** Study characteristics and outcomes of systematic review.

Reference, Year	Intervention, Study Design	No. of Patients	Treated Levels	Follow-Up	Outcome	Complications, Reoperations
De Rooij et al., 2022 [6]	PCN, Retrospective	158	C4-Th1, 158	41.5 months	67.8% fully recovered, 93.3% satisfied	Reoperation rate: 21.4%
Kim et al., 2022 [7]	PCN, Retrospective	44	C4-C7, 44	15.4 months	VAS: 7.5 to 2.9 at last FU, 59.1% excellent/good (McNab)	Reoperation rate: 3/44 (6.8%)
Pandolfi et al., 2021 [8]	PCN, Prospective	20	C3-C7, 22	2 years	VAS: 7.9 to 2.5 (2 years)	None
Li et al., 2020 [9]	PCN, Retrospective	40	C4-C6, 42	6 years	Clinical effective rates: 67.5–52.94% (short- to long-term FU)	Reoperation rate: 8.82%
Halim et al., 2013 [10]	PCN, Retrospective	69 Group A: 27 (fulfilled ideal selection criteria). Group B: 42	Group A: C3-C7, 27 Group B: C3-C7, 50	24 months	Pain relief: 78% in Group A, 60% in Group B. No improvement/worse pain: 22% in Group A, 38% in Group B	None
Sim et al., 2011 [11]	PCN, Retrospective	22	C3-C7, 46	6 months	VAS: 9.3 to 3.4. McNab: excellent/good 17/22 (77.3%)	None
Timmermann et al., 2011 [12]	PCN, Prospective	32	C2-C7, 40	4–6 weeks	VAS: 8.8 to 0.4 at 4 weeks	None
Li et al., 2008 [13]	PCN, Prospective	126	C3-C7, 125	2 years	MacNab excellent/good: 103/126 (83.7%)	Perc-D Spine Wand broken in one patient
Bonaldi et al., 2006 [14]	PCN, Prospective	55	C4-C7, 75	2–29 months	McNab: excellent/good 80–85%	One discitis, rupture of the tip of Perc-DC Spine Wand in one instance
He et al., 2020 [15]	PCA, Prospective	33	C4-C7, 33	1 year	VAS: 6.8 to 2.5. McNab excellent: 64–82%	None
Ierardi et al., 2020 [16]	PCN vs. PCD, Retrospective	50 (26 PCN, 24 PCD)	C3-C7, PCN: 26 PCD: 24	6 months	MacNab excellent/good: PCD 19/24 (79%), PCN 21/26 (81%) at 6 months	None
Li et al., 2019 [17]	PDCN (PCN + PCD), Retrospective	74	C3-C7, 130	1 year	Effective rates of 94.6% (1 week) and 90.6% (last FU). McNab: excellent/good 75.7% at last FU	2 cases of discitis (2.7%)
Yang et al., 2014 [18]	PCD vs. PCN vs. PCDN, Retrospective	171 (97 PCD,50 PCN, 24 PCDN)	C3-C7, PCD: 98, PCN: 50, PCDN: 23	2–8 years	McNab excellent/good: 81.3% (PCD), 82.4% (PCN), 83.2 (PCDN)	During PCN, plasma knife was broken. Discitis: 1 PCD,1 PCN
Schubert et al., 2014 [19]	PCD, Retrospective	95	C3-C7, 107	2 years	MacNab excellent/good: 81.4%; 81 patients (94.2%) would choose this method again	9 reoperations
Yan et al., 2010 [20]	PCN vs. PCD, Retrospective	176 (81 PCN, 95 PCD)	C3-C7, PCN: 81 PCD: 95	16–48 months	McNab excellent/good: 77.8% (PCN), 79.5% (PCD)	Perc-D Spine Wand broken after PCN; 1 discitis after PCD
Chen et al., 2022 [21]	PCN vs. CT, Prospective	71 PCN, 21 CT	C3-C7, 127	6 months	PCN significantly superior to CT for both pain relief and functional improvements	Re-surgery rate 4.44% (4/90) after PCN
Cesaroni et al., 2010 [22]	PCN vs. CT, RCT	62 PCN, 53 CT	Not reported	1 year	Significant VAS and NDI improvement in PCN group	None
Birnbaum, 2009 [23]	PCN vs. CT, RCT	26 PCN, 30 CT	C4-C7, 29	2 years	VAS: PCN 8.8 to 2.3, CT 8.4 to 5.1	None
Nardi et al., 2005 [24]	PCN vs. CT, RCT	50 PCN, 20 CT	C4-C7, 54	60 days	80% symptom resolution in PCN, 75% persistent pain in CT	4 reoperations
Halim, 2017 [25]	PCN vs. PRF, RCT	34 (17 PCN, 17 PRF)	PCN: C5-7, 17 PRF: C3-7, 17	3 months	VAS improvement: PCN 43.4 points, PRF 34.0 points	None
Rooij et al., 2020 [26]	PCN vs. ACD, RCT	48 (24 PCN, 24 ACD)	ACD: C4-C7, 24 PCN: C5-C7, 24	12 months	More improvement in arm pain in the ACD group	None

**Table 2:** Risk of bias evaluation using Cochrane risk of bias assessment tool (the 18 March 2021 version) for randomized trials (RoB 2).

RoB 2 Domains *						
RCT	1a	1b	2	3	4	5
Rooij et al., 2020 [26]	Some concerns	High risk	Some concerns	Low risk	Low risk	Low risk
Halim et al., 2017 [25]	Low risk	Low risk	Low risk	Low risk	Low risk	Low risk
Cesaroni et al., 2010 [22]	Low risk	Low risk	Low risk	Low risk	Low risk	Low risk
Birnbaum et al., 2009 [23]	Low risk	Low risk	Low risk	Low risk	Low risk	Low risk
Nardi et al., 2005 [24]	Some concerns	Some concerns	Low risk	Low risk	Low risk	Low risk

* RoB 2 Domains: 1a. randomization process; 1b. participants’ recruitment; 2. deviations from the intended interventions; 3. missing outcome data; 4. measurement of the outcomes; 5. selective reporting.

**Table 3:** Risk of bias evaluation according to the Cochrane risk of bias in nonrandomized studies of interventions (ROBINS-I) assessment tool.

	ROBINS-I Domains **						
NRS	1	2	3	4	5	6	7
De Rooij et al., 2022 [6]	Low risk	Low risk	Low risk	Low risk	Serious risk	Moderate risk	Low risk
Kim et al., 2022 [7]	Low risk	Moderate risk	Low risk	Low risk	Low risk	Low risk	Low risk
Chen et al., 2022 [21]	Low risk	Low risk	Low risk	Low risk	Moderate risk	Moderate risk	Low risk
Pandolfi et al., 2021 [8]	Low risk	Moderate risk	Moderate risk	Low risk	Moderate risk	Low risk	Low risk
Li et al., 2020 [9]	Low risk	Moderate risk	Low risk	Low risk	Moderate risk	Low risk	Low risk
He et al., 2020 [15]	Low risk	Low risk	Low risk	Low risk	Moderate risk	Low risk	Low risk
Ierardi et al., 2020 [16]	Low risk	Low risk	Moderate risk	Low risk	Low risk	Moderate risk	Low risk
Li et al., 2019 [17]	Moderate risk	Moderate risk	Low risk	Low risk	Serious risk	Low risk	Low risk
Yang et al., 2014 [18]	Low risk	Moderate risk	Low risk	Low risk	Low risk	Low risk	Low risk
Schubert et al., 2014 [19]	Low risk	Serious risk	Low risk	Low risk	Moderate risk	Low risk	Low risk
Halim et al., 2013 [10]	Moderate risk	Moderate risk	Low risk	Low risk	Moderate risk	Low risk	Low risk
Sim et al., 2011 [11]	Low risk	Moderate risk	Moderate risk	Low risk	Low risk	Low risk	Low risk
Timmermann et al., 2011 [12]	Moderate risk	Moderate risk	Low risk	Moderate risk	Serious risk	Moderate risk	Low risk
Yan et al., 2010 [20]	Low risk	Low risk	Low risk	Low risk	Moderate risk	Low risk	Low risk
Li et al., 2008 [13]	Low risk	Low risk	Low risk	Low risk	Low risk	Low risk	Low risk
Bonaldi et al., 2006 [14]	Low risk	Moderate risk	Moderate risk	Low risk	Low risk	Low risk	Low risk

** ROBINS-I Domains: 1. baseline confounders; 2. participants’ enrollment; 3. interventions’ classification; 4. deviation from intended interventions; 5. incomplete outcome data; 6. measurement of the outcomes; 7. selective reporting. NRS—nonrandomized study.

**Table 4:** Comprehensive subgroup analysis.

Subgroup	Studies Included	Hedges’g	95% CI	Interpretation
Short-term follow-up	Sim, 2011 [11]	2.98	2.01–3.95	Very large short-term effect
	Timmermann, 2011 [12]	3.57	2.99–4.15	Very large, short-term effect
Long-term follow-up	De Rooij, 2022 [6]	0.0	−0.15–0.15	No long-term effect
	Kim, 2022 [7]	1.93	1.43–2.43	Moderate-to-large long term effect
	Pandolfi, 2021 [8]	2.36	1.46–3.27	Large long-term effect
	Li, 2020 [9]	−1.77	−2.26–−1.28	Negative long-term effect
Prospective studies	Pandolfi, 2021 [8]	3.28	2.44–4.11	Strong effect in prospective studies
	Timmermann, 2011 [12]			
Retrospective studies	De Rooij, 2022 [6]	0.07	−0.07–0.21	Minimal effect in restrospective studies
	Kim, 2022 [7]			
	Li, 2020 [9]			
	Sim, 2011 [11]			

**Table 5:** Indications and contraindications for minimally invasive percutaneous surgical techniques due to the literature review.

Surgical Technique	Indications	Contraindications
Percutaneous Cervical Nucleoplasty (PCN)	Cervical radicular pain caused by contained soft disc herniation [6,7,9,13,21,26] MRI-confirmed foraminal or central disc protrusion without extrusion or sequestration [7,9,13] Persistent symptoms >6 weeks despite conservative treatment [6,8,24] Cervical vertigo associated with degenerative disc disease [9,17] Discogenic neck pain without neurologic deficits [6,10,13]	Extruded or migrated disc fragments [13,20] Severe spinal canal stenosis or myelopathy [7,10] Segmental instability or previous fusion surgery at the treated level [9,14] Infection, coagulopathy, or poor general condition for intervention [6,9] Large osteophytes or uncinate hypertrophy causing fixed compression [18,20]
Percutaneous Cervical Discectomy (PCD)	Contained or slightly extruded cervical disc herniation with radiculopathy [12,16,18,19] Unilateral or bilateral nerve root compression with corresponding clinical findings [18,19,20] Soft herniated nucleus pulposus confirmed on MRI [14,20] Failure of ≥6 weeks of non-operative treatment [12,19] Preference for minimally invasive technique over open discectomy [12,16,20]	Hard calcified discs or advanced degenerative disc disease [14,18] Large disc extrusion with sequestration [12,18] Severe myelopathy or spinal cord compression [16,20] Instability, severe disc height loss, or deformity [16] Infections, bleeding disorders, or inability to cooperate [12,14]
Percutaneous Cervical Annuloplasty (PCA)	Discogenic neck pain without radiculopathy, associated with annular fissures or internal disc disruption High-intensity zone (HIZ) on T2-weighted MRI at symptomatic level Positive provocative discography indicating annular pain source Persistent axial neck pain unresponsive to conservative management [15]	Radicular symptoms due to nerve root compression Disc extrusion or sequestration Severe cervical stenosis, myelopathy, or segmental instability Active systemic or local infection, coagulopathy, or pregnancy [15]
Percutaneous Cervical Pulsed Radiofrequency (PRF)	Cervical radicular pain due to contained disc herniation, especially when surgery is not indicated Chronic neuropathic pain involving dorsal root ganglion (DRG), including post-discectomy syndromes Patients non-responsive to medications, physical therapy, or steroid injections Alternative for patients unsuitable for surgical decompression [25]	Severe mechanical compression requiring surgical decompression Myelopathy or large extruded discs Local infection, systemic illness, or bleeding diathesis Implanted cardiac pacemakers or other electronic implants (relative contraindication) [25]

**Table 6:** Characteristics of the main demographic and FU parameters.

Study	*N*	Males, *n* (%)	Age, Years	FU, Months
De Rooij et al., 2022 [6]	158	74 (46.8%)	47.3 ± 9.1	41.5
Kim et al., 2022 [7]	44	27 (61.4%)	54.5 (range: 31.0–81.0)	15.4 (range: 3.7–30.8)
Pandolfi et al., 2021 [8]	18	10 (55.6%)	52.5	24.0
Sim et al., 2011 [11]	22	15 (68.2%)	47.8 ± 11.9	6.0
Timmermann et al., 2011 [12]	32	7 (21.9%)	59.1 ± 13.3	1.0–1.5
Li et al., 2008 [13]	126	65 (51.6%)	51.9 ± 10.2	24.0 (range: 14.0–36.0)

*N*—sample size; *n*—group size.

## Data Availability

Data are contained within the article.

## Supplementary Materials

The following supporting information can be downloaded at: https://www.mdpi.com/article/10.3390/jcm14103280/s1, Table S1: PRISMA 2020 Checklist. Reference [27] is cited in the Supplementary Materials.

## Abbreviations

ACD	Anterior Cervical Discectomy
ACDF	Anterior Cervical Discectomy and Fusion
CI	Confidence Interval
CT	Conservative Treatment
FU	Follow-up Period
NDI	Neck Disability Index
PCA	Percutaneous Cervical Annuloplasty
PCD	Percutaneous Cervical Discectomy
PCN	Percutaneous Cervical Nucleoplasty
PDCN	Percutaneous Cervical Discectomy with Nucleoplasty
PRF	Percutaneous Pulsed Radiofrequency
PRISMA	Preferred Reporting Items for Systematic Reviews and Meta-Analysis Guidelines
SMD	Standardised Mean Difference
VAS	Visual Analogue Scale
